# When “I” or “S/He” uses the product: the impact of narrative perspective on consumers’ brand attitudes in storytelling ads

**DOI:** 10.3389/fpsyg.2024.1338249

**Published:** 2024-07-12

**Authors:** Tingting Chen, Xinqiao Fan, Jiayu He, Jun Fan, Wei Chen

**Affiliations:** ^1^Business & Tourism Institute, Hangzhou Vocational & Technical College, Hangzhou, China; ^2^School of Business Administration, Zhejiang Gongshang University, Hangzhou, China

**Keywords:** story advertisement, narrative perspective, social presence, product involvement, brand attitude

## Abstract

**Introduction:**

Storytelling ad is presented from one or more narrative perspectives. Narrative perspective, which can alter the way in which the plot is physiologically or psychologically perceived, can significantly affect consumer experience.

**Methods:**

This study conducts three experiments with 526 participants to analyze the influencing mechanism of narrative perspective (first- versus third-person) on consumers’ brand attitudes in storytelling ads of products with different involvement (high versus low).

**Results:**

(a) Narrative perspective (first- versus third-person) exerts persuasive effects on consumer brand attitudes; (b) Processes of social presence and self-brand connection explain the effects of narrative perspective on brand attitudes; (c) When product involvement is high, the use of the first-person narrative perspective in storytelling ads will result in a more positive brand attitude than the use of third-person narrative will; With lower product involvement, there is no significant difference in the impact on brand attitudes regardless of narrative perspective (first-person versus third-person).

**Discussion:**

This research finds that different narrative perspectives significantly impact the persuasiveness of advertising. Boundary conditions exist for the effect of narrative persuasion, and product involvement moderates the effect of narrative perspective on brand attitudes.

## 1 Introduction

The proliferation of video applications, social media platforms, and e-commerce portals has rendered video watching and sharing a predominant mode for consumers to acquire information, entertainment, and engage in social interaction during leisure hours. Conventional advertising, reliant on text, imagery, and sound, fails to adequately satisfy contemporary sensory consumer demands. In contrast, storytelling advertisements adopt a subtler marketing strategy, embedding brand messaging within a plot plus brand’ format that fosters consumer immersion in the story and gradual receptiveness to brand influence (Fan and Pan, 2016). Such advertisements narrate tales of product use or affiliated experiences, or depict product consequences, conveying the narrator’s responses to events, setting scenes, and timelines, captivating audiences, and thereby achieving persuasive ends (Ching et al., 2013).

Narrative perspective in storytelling ads, capable of altering the psychological or emotional absorption of the storyline, exerts a significant impact on consumer experience (Segal et al., 1997; Kim et al., 2019). This perspective enables consumers to perceive characters’ thoughts and emotions, engendering rich sensory and emotional engagement, and fostering a sense of immediacy and relevance, ultimately stimulating favorable brand attitudes (De Graaf et al., 2012; Kim et al., 2014, 2017). Two principal narrative viewpoints exist: the first-person perspective, where the protagonist narrates, and the third-person perspective, employing an outsider narrator (Segal et al., 1997; Feng et al., 2019). Both are widely employed in practice, warranting investigation into which perspective proves more persuasive and influential in cultivating positive brand sentiment.

Narrative perspective constitutes the fundamental narrative attribute of a story, dictating the primary mode of audience communication (Kim et al., 2016). Narrators can either embody a character within the story (first-person) or operate as external observers unaffiliated with any character (third-person; Davis et al., 2004; Pachucki et al., 2022). Prior research on narrative perspective, though prevalent in communication (De Graaf et al., 2012), news (Kim et al., 2019), and tourism (Pachucki et al., 2022), is scarce in the context of story-based advertisements, particularly video ads, with incomplete understanding of its impact mechanisms and a narrow range of application scenarios. Commonly agreed upon is the role of perceived presence in narrative effects generation (Kim et al., 2019; Pachucki et al., 2022), with select dimensions potentially more pivotal for persuasion (De Graaf et al., 2012). Some theorists posit self-brand connection as the underlying mechanism for narrative persuasion (Lim and Childs, 2020), yet a causal examination of social presence and self-brand connection on brand attitudes remains lacking. This study zeros in on these dimensions.

Despite prior research indicating that different narrative perspectives significantly impact persuasiveness, a detailed mechanism of this effect requires clarification, and potential interference from additional information must be considered (De Graaf et al., 2012; Hoeken et al., 2016; Kim et al., 2019). Researchers like Segal et al. (1997) and Winterbottom et al. (2008) contend that first-person narrative perspective is more convincing than third-person because it fosters a closer relationship between the audience and the protagonist (Segal et al., 1997). Nonetheless, this conclusion is not universally true across all circumstances, particularly when the protagonist is viewed as an in-group member as opposed to an out-group member (Banerjee and Greene, 2012; Kaufman and Libby, 2012). Moreover, when the audience is in an analytical mindset, third-person narratives prove more persuasive than first-person ones (Kim and Shapiro, 2016). Furthermore, the level of product involvement influences the persuasive effectiveness of advertising narratives (Lien and Chen, 2013; Kim et al., 2017). Notably, past studies investigating narrative persuasion’s impact on product involvement have overlooked the effects of prior knowledge and product characteristics (Petty et al., 1986; Kim et al., 2017). The manipulation of product involvement in our study aligns with Petty et al.’s framework, preserving the receiver’s, information’s, and media’s characteristics unchanged (Petty et al., 1986).

Adopting the Elaboration Likelihood Model and focusing on social presence and self-brand connection, this research conducts three experiments to explore the mechanism by which narrative perspective (first-person vs. third-person) influences consumers’ brand attitudes under varying degrees of product involvement (high vs. low), achieved through manipulating the narrative voice in video advertisements. It contributes to the body of knowledge on storytelling advertising and deepens theoretical explorations of how consumers process video messages in advertisements. By unraveling the black box of persuasive mechanisms linked to narrative perspective and defining boundary conditions in video ads, this study not only enriches academic understanding but also provides practical insights for companies seeking to employ storytelling ads to effectively enhance consumer-brand relationships.

## 2 Literature review and hypotheses

### 2.1 The elaboration likelihood model and product involvement

The Elaboration Likelihood Model (ELM) is a comprehensive theory explicating attitude change, furnishing a thorough framework to decipher the fundamental mechanisms underlying persuasive effectiveness (Petty and Cacioppo, 1986). It delineates two pathways of persuasion during cognitive information processing: the central and peripheral routes (Petty and Cacioppo, 1984, 1986). The first, central route, entails a deliberate and thoughtful assessment of the actual merits of information advocating for a position. Conversely, the peripheral route offers a swift means of accepting or rejecting information without deeply engaging with the message’s inherent qualities (Petty and Cacioppo, 1986). Under this route, consumers tend to process information superficially, conserving cognitive resources and minimizing mental effort.

Product involvement can be understood as the subjective relevance of a product category to an individual consumer, derived from their personal values, needs, and interests (Lastovicka, 1979; Zaichkowsky, 1994; Kim et al., 2017). Given the substantial variance in information processing styles influenced by product involvement, this concept has been extensively employed as an explanatory factor in consumer behavior research (Zaichkowsky, 1994). Our study adopts the conceptualization of product involvement as outlined by Lastovicka (1979) and Zaichkowsky (1994).

Extensive research has evidenced the impact of product involvement on a spectrum of behavioral outcomes, including information seeking and processing behaviors (Richins and Bloch, 1986). In consumer behavior, the level of product involvement is manifested in the complexity of cognitive and behavioral processes engaged. High product involvement prompts consumers to predominantly utilize the central route, whereas low involvement situations see consumers adopting the peripheral route (Petty et al., 1983; Petty and Cacioppo, 1984). Ad viewing, where consumers glean and interpret product information, can be construed as an attitude transformation process. By elucidating attitude change through both central and peripheral routes, ELM furnishes a robust theoretical foundation for our investigation, enhancing its explanatory power and clarity.

### 2.2 Narrative perspective

Narrative perspective is a term used to describe the narrator of a story. Each story has a unique viewing position, primarily in the first person and third person (Banerjee and Greene, 2012). From the perspective of the first person, the story usually begins with the narrator as the protagonist, whose inner thoughts and personal experiences can be candidly conveyed to consumers. From the perspective of the third person, a narrator is an unspecified person who conveys the story but may not be a character in the story (Segal et al., 1997). In terms of language, the narrative perspective is constructed from two dimensions: the relationship between the narrator and behavior, and the degree to which the narrator observes the thoughts and behaviors of the characters (Gu and Tse, 2016). The former is usually labeled as ‘point of view,’ which is primarily based on the different pronouns used by the narrator (for example, I vs. he/she/them). The first-person narrative casts the narrator as the main communicator (e.g., by referring to ‘my camera’), while the third-person narrator tells the story of an individual from the perspective of an onlooker (e.g., ‘Zhang Jie’s camera’; Gu and Tse, 2016; Kim et al., 2019). The latter is called internal or external focalization, which is mainly inferred by describing of characters’ the inner thoughts and emotions (Peer and Maat, 2001; Kaufman and Libby, 2012). This research follows Segal et al. (1997) and Banerjee and Greene (2012) on the concept of the narrative perspective.

### 2.3 Social presence

Social presence denotes the extent to which individuals perceive the existence of others in communicative exchanges, encapsulating whether individuals can establish a sense of intimacy or immediacy in interpersonal interactions (Lu et al., 2016; Fan et al., 2019). Rooted in social psychological theories of interpersonal communication and symbolic interactionism, social presence theory is prominently featured in discussions surrounding mediated communication contexts (Cui et al., 2013; Kofi and Graeme, 2018). When a narrative’s protagonist or character is perceived as a ‘real entity,’ interactions ensue as though devoid of any mediating technology, thereby fostering a sensation of ‘being present’ within the narrative setting (Biocca, 2002; Yan and Yang, 2013). This perception bridges the gap between the audience and the narrative world, heightening engagement and the sense of authenticity in the communication experience.

### 2.4 Self-brand connection

Self-brand connection concerns the bond between a brand and consumers’ identity. Consumers incorporate brand affiliations (such as user image and personality) into their own selves, linking brand association with their self-image in their minds (Escalas, 2004; Du et al., 2009). The greater the significance of a brand to consumers, the closer the relationship between the brand and the consumers becomes (Escalas, 2004). Self-brand connections can be fostered through advertising, social popular culture, and the consumers’ personal experiences with the brand. Narrative processing can further create or enhance consumer self-brand connections, as consumers tend to integrate their own experiences into a brand story to derive or explain their own meanings (Escalas, 2004; Lien and Chen, 2013). This study adopts the definition of self-brand connection from Escalas (2004) and Du et al. (2009).

#### 2.4.1 The influence of narrative perspective on brand attitude

Brand attitude denotes the internal psychological evaluation of a brand formed by consumers, primarily rooted in their previous experiences with the brand and the emphasis on brand attributes. Within the realm of advertising, consumer brand attitude serves as a crucial manifestation of narrative persuasion’s efficacy. Empirical evidence suggests that narrative advertising outperforms traditional non-narrative approaches in terms of persuasive effectiveness, as it fosters a more favorable disposition toward the brand among consumers (Chang, 2013; Kim et al., 2017). Furthermore, the narrative perspective adopted in advertisements exerts a significant influence on persuasive outcomes, with alterations in perspective capable of directly modifying consumer brand attitudes (De Graaf et al., 2012; Hoeken et al., 2016).

Banerjee and Greene (2012) highlight that information processing by viewers differs when comparing first-person vs. third-person narrative perspectives, which in turn affects the persuasiveness of the narrative. Complementing this, Brunyé et al. (2009) contend that narratives told from a first-person perspective tend to be more persuasive to audiences compared to those recounted in the third person. Grounded in these observations, we posit the following hypothesis:

*H1*: Using the first-person narrative perspective in storytelling ads will result in a more positive brand attitude than the use of third-person narrative perspective.

### 2.5 Mediating effect of social presence

When audience members become absorbed in a narrative, the story engenders a palpable sense of presence, which in turn modifies their attitudes and beliefs (Li et al., 2015). Narrative techniques facilitate the integration of the audience’s experiences into the storyline, simulating the protagonist’s role and fostering mental social interactions and a virtual social milieu, thereby augmenting social presence (Mar and Oatley, 2008; Kaufman and Libby, 2012). Social presence theory posits that this heightened sense of presence promotes a favorable audience disposition toward the protagonist or characters (Lee and Jang, 2013). Social presence, as defined, implies the authentic existence of a role that cohabits the same social space as the audience, prompting the audience to attentively engage with the role’s thoughts and emotions (Biocca, 2002; Kim et al., 2019). Even when audiences recognize that narrative is fictional, the immersive experience and emotional closeness to the character fosters a positive attitude toward the protagonist or character (Kaufman and Libby, 2012; Li et al., 2015).

In comparison to the third-person narrative, the first-person perspective allows for direct social interaction from the protagonist’s vantage point, enabling consumers to intuitively immerse themselves in the protagonist’s psyche and live through product/brand-related narratives. This intensifies the sense of social presence among consumers, subsequently leading to a more positive outlook toward the product/brand through the advertisement. Consequently, we propose the following hypothesis:

*H*2: Social presence will mediate the relationship between narrative perspective and brand attitude.

### 2.6 Mediating effect of self-brand connection

People naturally prefer to imagine in story form. Narrative advertising can guide consumers to connect themselves conceptually with the brand and products being advertised, to give more meaning to the brand and make brands a means for consumers to form self-concepts (Escalas, 2004). After viewing narrative ads, consumers connect their previous experiences, memories, ideas, and subjective knowledge with the brand information in the ads to build a new story. This helps in forming or strengthening an imaginary connection between the self-concepts of the consumers and the product/brand. This type of self-brand connection can promote positive brand attitudes (Escalas, 2004; Du et al., 2009). Simultaneously, different narrative perspectives can also affect self-brand connections; For example, ‘in-group’ narrative perspectives produce stronger self-brand connections than ‘out-group’ perspectives (Moore and Homer, 2008). Previous studies have found that being transported into a branded story facilitates a self-brand connection (Escalas, 2004; Lim and Childs, 2020). When consumers generate self-brand connection, consumers believe that the concept of brand is a part of themselves (Lim and Childs, 2020). Therefore, they will prefer it over others (Thomson et al., 2005).

Compared with the third-person narrative perspective, the first-person narrative perspective creates a sense of intimacy between consumers and the protagonists in the ads (Brunyé et al., 2009). This kind of closeness effortlessly enables consumers to incorporate the image and personality of the protagonist in the story, and promote the connection between brand association and self-image, thereby ensuring consumers have a positive attitude similar to the protagonist (Escalas, 2004). Therefore, we hypothesize that:

*H*3: Self-brand connection plays a mediating role in the influence of the narrative perspective on brand attitude.

### 2.7 The influence of narrative perspective on brand attitude in different product involvement

The persuasiveness of different narrative perspectives is contingent upon the processing methods utilized (Kaufman and Libby, 2012). Product involvement influences consumers’ motivation and the persuasive pathway they take when processing advertising information (Jin, 2009). Generally, with high involvement products, consumers tend to process product information more meticulously and comprehensively; conversely, with low involvement products, they often base their judgments on simple cues (Wang et al., 2018). Petty and Cacioppo (1986) note that under high involvement conditions, the central route to persuasion is adopted, which posits that attitude change results from careful consideration. In low involvement situations, the peripheral route is favored (Jin, 2009). Consumers with low involvement are likely to minimize effort and allocate fewer cognitive resources (Petty et al., 2002).

As a form of narrative advertising, storytelling ads also demonstrate their persuasive effect through the positive brand attitudes they cultivate in consumers (Yuan et al., 2010; Kim et al., 2019). When the level of product involvement is high, consumers perceive the product as significant and pay close attention to advertising (Zaichkowsky, 1994; Baumeister and Finkel, 2010; Kim et al., 2017). Drawing from the Elaboration Likelihood Model (ELM), it is argued that consumers who follow the central route will give greater attention to information, such as stories, related to product information in ads. At this juncture, the first-person narrative, compared to the third person, is likely to elicit more positive brand attitudes in consumers. In contrast, when product involvement is low, consumers take a peripheral route to analyze the product and pay less attention to the story. As a result, there is no significant difference in the impact of narrative perspective on the brand attitude of the product. Based on these considerations, we propose the following hypotheses:

*H*4: When product involvement is high, the use of the first-person narrative perspective in storytelling ads will result in a more positive brand attitude than the use of third-person narrative perspective.

*H*5: When product involvement is low, narrative perspective of storytelling ads has no significant effect on brand attitude.

**Figure 1:**
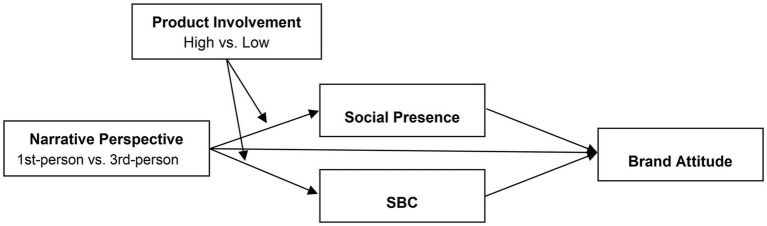
Conceptual model.

Based on the above theoretical analysis and research hypotheses, the conceptual model for this study is constructed, as presented in Figure 1.

## 3 The influence of the narrative perspective (first-person vs. third-person) on the brand attitudes of consumers (experiment 1)

The purpose of this experiment is to examine the influence of the narrative perspective (first-person vs. third-person) on the brand attitudes of consumers.

### 3.1 Method

**Materials**. In experiment 1, a camera brand that is a fictitious brand, PAPER, was used to eliminate the possible influence of existing consumer brand attitudes. The two video ads used in the formal experiment have different narrative perspectives, and the rest (such as plot, recording elements, editing methods) remain unaltered. The narrator’s voice is the same, but the narrative perspective differs. The video duration is approximately 1.5 min. The ads describe that the protagonist (Zhang Jie) always uses her camera to record unforgettable events (fragments) in her life, such as love, marriage, the birth of children, old age life, and so on.

The advertising narrative perspective is mainly manipulated by describing Zhang Jie’s story with different narrative perspectives (voice), namely, the first person (I, video 1, 125 words) and the third person (she, video 2, 135 words).

- Video 1 (First-Person Perspective): Zhang Jie tells her (my) story (for example, ‘when I was 16 years old, I used my first PAPER camera to record moments in my life’).
- Video 2 (Third-Person Perspective): The story of Zhang Jie (she) is narrated from the perspective of an external onlooker (for example, ‘when Zhang Jie was 16 years old, she used her first PAPER camera to record moments in her life’).

**Manipulation check of the narrative perspective**. In Experiment 1, 30 college students were invited to participate in the pretest. To ensure that the advertising video material can be played smoothly, the video is uploaded to the third-party video playing website without advertising. After watching the video ads, the subjects were asked to make a subjective judgment on the narrative perspective of the video ads. The question is: What perspective do you think the ads use to narrate? The results showed that 100% of the subjects chose correctly. Thus, the manipulation of the narrative perspective in experiment 1 is successful.

**Participants and procedure**. We published the questionnaire on Wenjuanxing, the largest professional data collection website in China.^1^ This website has more than one million participants answering questionnaires every day, and provides professional questionnaire collection services. The specific method is to upload the experimental video material to the video playing platform without website ads.^2^ And then embed the video at the beginning of the online questionnaire. The questionnaire was set to be completed after watching the experimental video. The questionnaire included two questions to identify invalid responses: both were similarly worded to two genuine items in the questionnaire but had opposite meanings. If participants gave the same answers to these two questions as their answers to the two genuine items, their questionnaire was classified as invalid. Presents the demographic information of the study’s participants. The sample size refers to previous experimental design studies (De Graaf et al., 2012; Lien and Chen, 2013; Fan and Pan, 2016; Feng et al., 2019). A total of 180 Chinese adults were recruited from a Wenjuanxing to participate in a web-based experiment. Participants were randomly assigned to a first-person or third-person narrative group (single-factor, between-subjects design). After eliminating the invalid questionnaires, 150 valid questionnaires were obtained. Of all the participants (see Table 1), 50.7% are women (*n* = 76) and 49.3% are men (*n* = 74). Most of the participants are aged either 18–30 (*n* = 69, 46.0%) or 31–40 (*n* = 38, 25.3%), and most have a bachelor’s degree (*n* = 99, 66%).

**Table 1:** Demographics of participant.

Items		Experiment 1		Experiment 1		Experiment 1	
		Number	Percentage	Number	Percentage	Number	Percentage
Gender	Male	74	49.3%	70	50.7%	114	47.9%
	Female	76	50.7%	68	49.3%	124	52.1%
Age	under 18	19	12.7%	18	13.0%	25	10.5%
	18–30	69	46.0%	70	50.7%	145	60.9%
	31–40	38	25.3%	34	24.6%	59	24.8%
	41–50	20	13.3%	13	9.4%	8	3.4%
	51 or older	4	2.7%	3	2.2%	1	0.4%
Education level	Secondary school or below	26	17.3%	25	18.1%	43	18.1%
	Junior college	25	16.7%	29	21.0%	45	18.9%
	Bachelor	67	44.7%	71	51.4%	122	51.3%
	Master’s	30	20.0%	12	8.7%	23	9.7%
	PhD	2	1.3%	1	0.7%	5	2.1%

**Questionnaire**. Cronbach’s alpha is used to measure the internal consistency reliability of a set of scale or test items. Using a 7-point Likert scale (1 = strongly disagree, 7 = strongly agree), mainly referring to the research of Kokkinaki and Lunt (1999), brand attitudes were measured with the following four items (Cronbach’s α = 0.880): dislike/like; unappealing/appealing; unattractive/attractive; undesirable/desirable.

### 3.2 Results discussion

The results indicate that the main effect of the narrative perspective on consumer brand attitude was significant (*F* = 2.065, *p* = 0.000 < 0.05). Furthermore, an independent sample t-test was used to analyze the influence of different narrative perspectives (first-person vs. third-person) on brand attitudes. The results demonstrate that (see Table 2), compared to the third-person narrative perspective, consumers had a more positive attitude toward the brand when the ads were presented in the first-person narrative (M_1st-person_ = 5.08, SD = 1.11 vs. M3rd-person = 3.99, SD = 1.20; *t* = 5.81, *p* = 0.000 < 0.05). Thus, hypothesis H1 is supported. (NOTE: The mean serves as a measure of central tendency, representing the average value of a dataset. The F-test is employed to assess the overall significance of the model, while the t-test is used to determine if there is a significant difference between the means of two groups).

**Table 2:** The impact of narrative perspectives on brand attitudes (Experiment 1).

Narrative role	Mean	SD	F	Sig
First-person	5.08	1.11	2.065	0.000***
Third-person	3.99	1.20		

^***^p < 0.001.

## 4 The mediating effect of social presence and self-brand association (experiment 2)

The purpose of this experiment is to examine the mediating effect of social presence and self-brand association.

### 4.1 Method

**Materials**. A chocolate brand that is a fictitious brand, YOU FU, was used to eliminate the possible influence of existing consumer brand attitudes and prevent the influence of self-brand connection of existing brands. The word ‘FU’ means luck in China. The advertisement tells the story that the protagonist named Zhang Jie is pursuing her dream and has not come home for many years. Her mother hopes to be reunited with her and can write the word ‘FU’ and eat ‘YOU FU’ chocolate with her.

The advertising narrative perspective is mainly manipulated by describing Zhang Jie’s story with different narrative perspectives (voice), namely, the first person (I, video 1, 210 words) and the third person (she, video 2, 210 words).

- Video 1: From the first-person narrative perspective, Zhang Jie tells her (my) story (for example, ‘I’m Zhang Jie. Every new year, my mother will write the word of ‘FU’. We will say ‘FU’ to each other every year and eat ‘YOU FU’ chocolate’).
- Video 2: In the third-person narrative perspective, the story of Zhang Jie (she) is narrated from the perspective of an external onlooker (for example, her name is Zhang Jie. Every new year, her mother will write the word ‘FU’. They will say ‘FU’ to each other every year and eat ‘YOU FU’ chocolate).

**Manipulation check of the narrative perspective**. Same as experiment 1. 30 college students were invited to participate in the pretest. Result shows that 100% of the subjects chose correctly, and the manipulation of narrative perspective in experiment 2 is successful.

**Participants and procedure**. A total of 180 Chinese adults were recruited from a Wenjuanxing to participate in a web-based experiment. After eliminating the invalid questionnaires, 138 valid questionnaires were obtained. Of all the participants (see Table 1), 49.3% are women (*n* = 68) and 50.7% are men (*n* = 70). Most of the participants are aged either 18–30 (*n* = 70, 50.7%) or 31–40 (*n* = 34, 24.6%), and most have a bachelor’s degree (*n* = 84, 60.9%).

**Questionnaire**. Using a 7-point Likert scale (1 = strongly disagree, 7 = strongly agree), The measurement of brand attitude refers to experiment 1 (Cronbach’s α = 0.908). Social presence was measured based on the research of Lee and Shin (2014), with the following four items (Cronbach’s α = 0.925): I felt as if I were engaging in an actual conversation with the narrator; I felt like I was in the same room as the narrator; I felt as if the narrator were speaking directly to me; I could imagine the narrator vividly. Self-brand connections were measured based on the research of Escalas and Bettman (2003), and the following seven items were measured (Cronbach’s α = 0.953): Brand X reflects who I am; I identify with the brand; I feel a personal connection to Brand X; I (can) use Brand X to communicate who I am to other people; I think Brand X (could) help(s) me become the type of person I want to be; I consider Brand X to be ‘me’ (it reflects who I consider myself to be or the way that I want to present myself to others); and Brand X suits me well.

### 4.2 Results discussion

The results indicate that the main effect of the narrative perspective on consumer brand attitude was significant (*F* = 14.474, *p* = 0.000 < 0.05). Furthermore, an independent sample t-test was used to analyze the influence of different narrative perspectives (first- vs. third-person) on consumer brand attitudes. The results indicate that (Table 3), compared with the third-person narrative perspective, consumers had a more positive brand attitude when presented with the ads that was from the first-person narrative perspective (M_1st-person_ = 5.39, SD = 0.82 vs. M_3rd-person_ = 3.74, SD = 1.28; *t* = −8.98, *p* = 0.000 < 0.05); Thus, hypothesis H1 has again been supported.

**Table 3:** The effect of different narrative perspectives on brand attitudes (Experiment 2).

Narrative role	Mean	SD	F	Sig
First-person	5.39	0.82	14.474	0.000***
Third-person	3.74	1.28		

^***^p < 0.001.

Based on the mediating effect analysis procedure proposed by Chen et al. (2013), the multiple parallel mediating variable test methods proposed by Preacher and Hayes (2008), and using bootstrapping in the process, the mediating effect of social presence and self-brand connection was evaluated. A mediation analysis was performed using PROCESS (Model 4 with 5,000 iterations); Preacher and Hayes (2008) with narrative perspective as the independent variable (coded 1 for the first-person and 0 for the third-person storytelling ad), social presence and self-brand connection as the mediators, and brand attitude as the dependent variable. Gender, age, and education level were included as covariates.

The co-mediation effect was significant (LLCI = 0.5644, ULCI = 1.2006), and the effect size was 0.8551. Of the two mediating pathways, social presence (LLCI = 0.1611, ULCI = 0.5413) and self-brand connection (LLCI = 0.3044, ULCI = 0.8396) had significant mediating effects, with effect sizes of 0.3081 and 0.5470, *p* < 0.001. These results show that H2 and H3 are all supported.

## 5 The moderating role of product involvement in the influence of narrative perspective on consumer brand attitudes (experiment 3)

The purpose of this experiment is to examine the moderating role of product involvement (high vs. low) on the relationship between narrative perspective (first-person vs. third-person) and consumer brand attitudes.

### 5.1 Method

**Materials**. A fictional beverage brand named “Hetaolu” was used. Please see the 3 section for details on the experimental materials’ editing approach. The video duration is about 1.5 min. The advertisement tells the story that the protagonist named Zhang Jie is preparing for the college entrance examination. Zhang Jie’s mother provides Zhang Jie with delicious meals and drinks (brand name ‘hetaolu’) every day. Finally, she got a good score on the college entrance examination.

The advertising narrative perspective mainly describes Zhang Jie’s story through different narrative perspectives (voice), that is, the first person (I, video 1, 200 words) and the third person (she, video 2, 199 words).

- Video 1: from the perspective of first-person narration, Zhang Jie tells her (me) story (for example, ‘I’m Zhang Jie, a senior three students, about to face the first turning point of life, the college entrance examination’).
- Video 2: in the third-person narrative perspective, Zhang Jie’s story is told from the perspective of external onlookers (for example, ‘Zhang Jie, a senior three students, is about to face the first turning point of life, the college entrance examination’).

The manipulation design of narrative perspective refers to experiment 1. The manipulation design of product involvement refers to the research of Petty et al. (1983) This method avoids the interference of irrelevant factors on the experimental results and has been unanimously recognized by the researcher. In this experiment, for two groups of subjects, the experimental reward item is “Hetaolu” beverage, so they will be more involved in the beverage. In addition, they are informed that the beverage will be sold locally, which further improves the degree of involvement. On the contrary, the other two groups were told that the reward items were random gifts, and the drinks of this brand would not be promoted locally in the near future. Therefore, they pay little attention to the investment of beverage brands, and their product involvement is also low. That is, group 1 was the first person with high involvement; Group 2 was the third person with high involvement; Group 3 was the first person with low involvement; Group 4 was the third person with low involvement.

**Manipulation check of narrative perspective and product involvement**. In Experiment 3, 60 college students were invited to participate in the pre-test. Narrative perspective manipulation reference experiment 1. The manipulation results show that the subjects’ judgment of narrative perspective is accurate. The manipulation of product involvement is studied by Laurent and Kapferer (1985) and Jin et al. (2018), and consisted of five items: “This beverage is very important to me” “I would regret my choice if I am not satisfied with the beverage I purchased” “When I buy beverage, I usually choose carefully to make the right decision” “I value the functional (hedonic) value that this beverage brings me” and “I think this beverage can reflect the user’s personality or social status.” The manipulation of product involvement was successful. Compared with random gifts, the group with “Hetaolu” beverage had higher product involvement (*p* < 0.05).

**Participants and procedure**. The participants were invited to formal experiment. Using a between-participants design in experiment 3, participants were randomly assigned to 4 conditions. The process and method of experiment 3 are the same as experiment 1. A total of 270 questionnaires were sent out for new participants, and 238 valid questionnaires were obtained after eliminating the invalid questionnaires. Of all the participants (see Table 1), slightly more than half (*n* = 124, 52.1%) were females. Most of the participants are aged either 18–30 (*n* = 145, 60.9%) or 31–40 (*n* = 59, 24.8%), and most have a bachelor’s degree (*n* = 150, 63.0%). The items of brand attitude (Cronbach’s α = 0.923), self-brand connection (Cronbach’s α = 0.966) and social presence (Cronbach’s α = 0.908) are the same as experiment 2.

### 5.2 Results discussion

**Figure 2:**
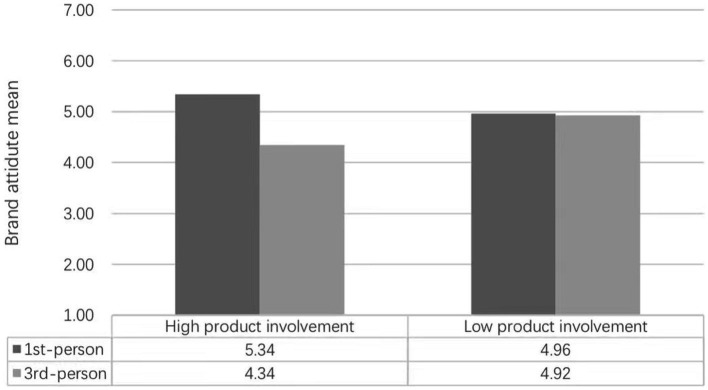
The result of brand attitude in experiment 3.

Brand attitude was the dependent variable, and social presence and self-brand connection were the intermediary variables. It can be found that the main effect of narrative perspective is significant, *F* = 6.87, *p* = 0.009 < 0.01, the impact of product involvement on consumers’ brand attitude is not significant, *F* = 0.30, *p* = 0.59 > 0.01, the interaction is significant, *F* = 7.97, *p* = 0.005 < 0.01. The data show that product involvement plays a regulatory role in the impact of advertising narrative perspective on consumers’ brand attitude. As shown in Figure 2, when the product involvement is high, compared with the third-person perspective narration of advertising, the first-person perspective narration of advertising is more likely to trigger consumers’ positive brand attitude (M_1st-person_ = 5.34, M_3rd-person_ = 4.34, *F* = 1.188, *p* = 0.001 < 0.05). When product involvement is low, there is no significant difference in the impact of the advertising narrative perspective on consumers’ brand attitude (M_1st-person_t = 4.96, M_3rd-person_ = 4.92, *F* = 0.79, *p* = 0.877 > 0.05). Hypothesis H1 has been effectively verified again, and hypothesis H4 and hypothesis H5 have also been verified.

For mediating effect test, the analysis results show that using process model 4, the sample size is 5,000. Under the 95% confidence interval, the co-mediation effect is significant (LLCI = 0.0685, ULCI = 0.6708), and the effect is 0.3710; In the two mediating paths, social presence (LLCI = 0.0257, ULCI = 0.3196) and self-brand connection (LLCI = 0.0204, ULCI = 0.4035) play a significant mediating role, with the effect of 0.1582 and 0.2128, respectively. It can be seen that hypothesis H2 and hypothesis H3 have been effectively verified again.

Further clarify the intermediary role of social presence and self-brand connection. Referring to the analytical procedures of Preacher et al. (2007), Chen and Wang (2015), Li and Zhang (2018), model 7 was selected for the moderated mediating effect test. As shown in Table 4, the results show that when the product involvement is high, social presence (LLCI = 0.1338, ULCI = 0.6295) plays a significant mediating role, and the mediating effect is 0.3545; Self-brand connection (LLCI = 0.1862, ULCI = 0.8212) played a significant mediating role, and the mediating effect of self-brand connection is 0.4687; When the product involvement is low, the confidence interval of self-brand connection includes 0. When the intermediary is social presence, the index is −0.3666, Boot se is 0.1585, and the confidence interval is (LLCI = −0.7242, ULCI = −0.0936). The index is −0.4764, Boot se is 0.2150, and the confidence interval is (LLCI = −0.9405, ULCI = −0.0873).

**Table 4:** Moderated mediation models: Experiment 3.

		Conditional indirect effects				Index of moderated mediation			
	PI	Effect	Bootse	BootLLCI	BootULCI	INDEX	Bootse	BootLLCI	BootULCI
SP	High	0.35	0.12	0.1338	0.6295	−0.37	0.16	−0.7242	−0.0936
	Low	−0.01	0.09	−0.2035	0.1538				
SBC	High	0.47	0.16	0.1862	0.8212	−0.48	0.21	−0.9405	−0.0873
	Low	−0.01	0.13	−0.2731	0.2476				

PI, product involvement; SP, Social presence; SBC, Self-brand connection.

## 6 Global discussion

The overarching findings across Experiments 1, 2 and 3 converge to highlight a pivotal insight in advertising narratives: the first-person perspective serves as a potent instrument for enhancing consumer brand attitudes within storytelling advertisements. This consistency in results underscores the robustness of the observed effect, where stories narrated in the first person foster a heightened sense of intimacy with the narrator among consumers. This intimacy, in turn, facilitates improved comprehension and receptivity to the conveyed message, ultimately yielding superior persuasive outcomes. As shown in Table 5, all hypotheses have been validated.

**Table 5:** Conclusion about the hypothesis.

Hypothesis	Confirmation/Not Rejection
H1: Using the first-person narrative perspective in storytelling ads will result in a more positive brand attitude than the use of third-person narrative perspective.	Confirm
H2: Social presence will mediate the relationship between narrative perspective and brand attitude.	Confirm
H3: Self-brand connection plays a mediating role in the influence of the narrative perspective on brand attitude.	Confirm
H4: When product involvement is high, the use of the first-person narrative perspective in storytelling ads will result in a more positive brand attitude than the use of third-person narrative perspective.	Confirm
H5: When product involvement is low, narrative perspective of storytelling ads has no significant effect on brand attitude.	Confirm

Our investigations contribute to the literature by concurrently examining and confirming the advantageous impact of first-person narratives across three distinct experiments, thereby enriching our understanding of narrative persuasion in advertising beyond what has been explored by prior studies such as those by De Graaf et al. (2012), Hoeken et al. (2016), Kim et al. (2019), Lim and Childs (2020), and Pachucki et al. (2022). Specifically, our work extends the boundaries of narrative perspective research by providing empirical evidence that supports the consistent advantage of first-person narratives across different contexts and measures.

Nonetheless, recognizing the necessity for a deeper exploration, we acknowledge several avenues for further refinement. While our experiments underscore the effectiveness of first-person narratives, they do not fully elucidate the underlying mechanisms, particularly the roles of social presence and self-brand connection, which may mediate the observed effects. Understanding these mediators would offer a more nuanced explanation of how narrative perspective influences consumer attitudes and behaviors.

Furthermore, we recognize the limitation of not initially addressing the potential moderating effect of product involvement, an oversight rectified in Experiment 3. By examining this moderating role, our study aims to provide a comprehensive picture of when and how narrative perspective interacts with product-related factors to shape consumer responses, thereby enhancing the practical implications for marketers targeting diverse product categories.

## 7 Conclusion

Existing studies have extensively discussed the persuasion effect of storytelling on advertising, and most of the studies have focused on text and picture ads (Phillips and McQuarrie, 2010; Kim et al., 2014, 2017; Lim and Childs, 2020). And existing research on the marketing effectiveness of storytelling ads lacks research on narrative perspective, mainly focusing on areas such as story format (Lien and Chen, 2013) and design elements (Ching et al., 2013). Past research on narrative perspective usually explores the mechanisms underlying narrative persuasion from the perspectives of identity, narrative transmission, and social presence, which cannot be applied to all research scenarios (De Graaf et al., 2012; Hoeken et al., 2016; Kim et al., 2019; Pachucki et al., 2022). Moreover, the discussion of the boundary conditions under which narrative persuasion holds is characterized by few studies and still inadequate discussion (Banerjee and Greene, 2012; Kaufman and Libby, 2012). The present study addresses these research gaps.

Our study’s main purpose is to investigate how narration persuades customers. For this purpose, we built a research model that emphasizes narrative perspective and brand attitude. Because the persuasive effect of narrative differs across product involvement, we proved our hypothesis through three experiments. The main conclusions are as follows. First, when the product involvement is high, compared to the third-person narrative perspective, the first-person narrative perspective can make consumers adopt a more positive brand attitude in storytelling ads. Social presence and self-brand connection play a mediating role, which complements and verifies the research results of Kim et al. (2019), Pachucki et al. (2022) Second, there is no significant difference between narrative perspective and brand attitude in storytelling ads with low product involvement, which is not supported by the research of Brunyé et al. (2009) and Kim et al. (2019) This may be because consumers adopt the peripheral persuasion route with low product involvement, and the narrative perspective of the story is not the focus of consumers.

## 8 Practical and managerial implications

The study has three main theoretical implications. Firstly, by adopting storytelling advertisements as the focal point of inquiry and leveraging the Elaboration Likelihood Model (ELM), this study examines the persuasive impact of such ads through the perspective of consumer brand attitude. This expansion broadens the horizons of video marketing research and supplements the understanding of storytelling ad’s effectiveness. Consumer brand attitude represents a pivotal indicator of advertising’s persuasiveness, yet prior studies within video marketing have primarily concentrated on aspects like video esthetics, interactivity, visual appeal, and communicative aspects, overlooking the link between storytelling ads and consumer brand attitudes.

Second, this study establishes product involvement as a crucial boundary condition for narrative perspective effects. Narrative advertising, such as storytelling ads, has a better persuasive effect than non-narrative advertising (Yan and Yang, 2013; Kim et al., 2019). However, the persuasive effect of storytelling ad is also influenced by numerous factors, such as narrative intensity (Lien and Chen, 2013), character similarity (Hoeken et al., 2016), narrative perspective (Kim and Shapiro, 2016), and product type (Kim et al., 2017), which have been neglected in previous studies.

Thirdly, this work introduces social presence and self-brand connections as innovative mechanisms elucidating how narrative perspectives (first-person vs. third-person) engender persuasive outcomes. The literature on narrative persuasion has hitherto overlooked the roles of social presence attributed to narrative characters and the establishment of self-brand connections within storytelling ads. This feeling of presence and connections is closely related to the virtual experience of characters involved in watching storytelling ads. This study further deepens the research of Escalas (2004), Lien and Chen (2013), Yan and Yang (2013), and Kim et al. (2017) and will be of value as a reference to researchers studying narrative advertising, such as storytelling ad.

The results of this study also provide managerial implications. An appropriate advertising narrative perspective should be selected based on the degree of consumer involvement with the product. Specifically, for products with high involvement, those with higher purchase and use risks, for which consumers pay more attention and need to apply more cognitive resources to make purchase decisions. The first-person narrative perspective is suitable to present brand stories to consumers. For products with low involvement, there is no need to pay attention to the narrative perspective in storytelling ads.

In addition to adopting a narrative perspective that matches the degree of product involvement as mentioned above. Advertisers should adopt various communication strategies, plot designs, and technical means, such as improving the integration of story plots and brand information, enriching storytelling methods and presentation means, strengthening the similarity and empathy between the protagonists and consumers, enhancing the tortuousness of the plot, optimizing video quality and playback effects, and increasing game and interactive elements. This integration enables consumers to focus on the story, generate immersive emotional and psychological experiences, and effectively connect brand association and self-image.

## 9 Limitations

The study encounters several limitations meriting consideration. Firstly, our investigation centered exclusively on the interplay between product involvement and narrative perspective in shaping consumer brand attitudes, excluding the potential influences of other variables such as brand characteristics, frame effects, story forms, plot content, consumer characteristics and character similarity, and so on. Secondly, while conducting the three experiments, we did not assess participants’ affection toward the presented stories. Affinity for the narratives could potentially sway consumers’ brand attitudes, and our failure to isolate the impact of story liking on the formation of positive attitudes constitutes a limitation. Thirdly, the assessment of advertising’s persuasive efficacy should encompass not merely shifts in consumer brand attitudes but also modifications and endurance of consumer behavior patterns, a dimension not thoroughly explored in the current research. Lastly, the research adopts a quantitative approach grounded in experimental design. Incorporating a qualitative phase preceding the quantitative analysis would enrich our conclusions by offering deeper insights. Nevertheless, due to the constraints of the experimental setting, our study confines its examination of storytelling ads’ persuasive impact to the realm of consumer brand attitude transformations. Future endeavors might overcome these limitations through mixed-methods research or expanded experimental designs to achieve a more holistic view.

## 10 Further research

In future research, we intend to consider the above problems and conduct a more in-depth, systematic theoretical, empirical analysis of storytelling ads. The following studies can be considered in the future: First, other scenarios of the persuasive effect of narrative perspective can be explored in the future. The scenarios of research in a narrative perspective are focused on communication research (De Graaf et al., 2012), news (Kim et al., 2019), tourism (Pachucki et al., 2022). It is valuable to explore the persuasive effect of narrative perspective in scenarios such as green food, virtual communities, short videos, and so on; Secondly, explore new divisional dimensions of narrative perspective. The current division of narrative perspective is mainly based on character, personal pronouns (Banerjee and Greene, 2012; Gu and Tse, 2016). The persuasive effect of narrative perspective can be explored from the perspective of a group, such as in-group and out-group; Thirdly, the influence of other mediators on the persuasive effect of narrative perspective can be explored. Currently, the mediating mechanisms in narrative persuasion are identification (De Graaf et al., 2012; Hoeken et al., 2016), transportation (Pachucki et al., 2022), social presence (Kim et al., 2019; Pachucki et al., 2022). In the future, the influence of variables such as imagination fluency, self-brand connection, and empathy can be considered. Fourth, whether the storyteller is an expert or a “normal” consumer has an impact on the effectiveness of the narrative persuasion can be explored in the future.

## Data availability statement

The raw data supporting the conclusions of this article will be made available by the authors, without undue reservation.

## Ethics statement

The studies involving humans were approved by Hangzhou Vocational & Technical College. The studies were conducted in accordance with the local legislation and institutional requirements. The participants provided their written informed consent to participate in this study.

## Author contributions

TC: Writing – original draft, Writing – review & editing, Conceptualization, Data curation, Funding acquisition, Investigation, Methodology. XF: Data curation, Formal analysis, Methodology, Writing – original draft, Writing – review & editing. JH: Data curation, Formal analysis, Methodology, Writing – original draft, Writing – review & editing. JF: Conceptualization, Funding acquisition, Supervision, Writing – original draft, Writing – review & editing. WC: Writing – review & editing, Supervision.

## Footnotes

^1^https://www.wjx.cn/

^2^www.bilibili.com
